# A Demonstration on Disseminated Sclerosis

**Published:** 1906-12-01

**Authors:** Wm. Aldren Turner

**Affiliations:** Physician Outpatients' Department, King's College Hospital, and the Hospital for the Paralysed and Epileptic, Queen Square, London


### Hospital Clinics.

### A DEMONSTRATION ON DISSEMINATED SCLEROSIS.

By WM. ALDREN TURNER, M.D.(Edin.), F.R.C.P.(Lond.), Physician Outpatients' Department, King's College Hospital, and the Hospital for the Paralysed and Epileptic, Queen Square, London.

Gentlemen,—I propose to-day to show you some illustrations of a common chronic disease of the nervous system which is frequent in young people, and although we are well acquainted with its symptomatology, our knowledge of its causation is still deficient. If one may believe statistics, the malady seems to be more common in this country than in America. We have at present several interesting and instructive cases of disseminated sclerosis in this hospital, and I propose to demonstrate a few examples of it clinically, and then to refer very briefly to the points by which it may be recognised and differentiated from another common nervous disorder.

Case 1.—This is a young woman, 22 years of age, with a history characteristic of the affection. At about 16 years of age, that is six years ago, she had an attack of cramps and numbness in the legs, which passed away. Four years ago she noticed some dimness of vision in the right eye, which lasted a while and then passed away. Two years ago she noticed weakness of the legs and a staggering gait, accompanied with some tremor of the arms, which also after a while passed away. A year ago she had a relapse, which lasted four months and then passed off. The present symptoms only date from the summer of this year. She has thus had four very distinct attacks with symptoms referable to the nervous system. From such a history one could almost assume the presence of disseminated sclerosis. Only one other malady is characterised by a history of this kind—namely, hysteria. On looking at the patient one notices a certain amount of instability. She is unable to walk without assistance. On putting the feet together and closing the eyes she presents in marked degree Romberg's symptom. There is slight hyper-extension of the fingers and hypotonus, almost as marked as in tabes. There is, however, no intention tremor, only a certain amount of awkwardness or jerkiness in voluntary movements. She has nystagmus on lateral deviation of the eyeballs to the left. She has also pale optic discs, an extensor plantar reflex; abdominal reflexes are absent. So that in addition to an ataxic gait, well-marked Romberg's symptom, and awkwardness of the hand movements, there is exaggeration of the knee-jerks, more particularly of the left, extensor plantar reflexes, and absence of abdominal reflexes. This patient, although she has been ill periodically for six years, is not in an advanced stage of the disease, because the symptoms she presents have only been in existence since June. It is well known that a person with disseminated sclerosis may have repeated paralysis, and yet the symptoms pass away until an attack sets in from which recovery does not take place.

Case 2.—This man is 35 years of age. He has dragging of the right foot, with some stiffness. His illness commenced two years ago with weakness and stiffness in the left foot and leg. A year ago the rigidity of both limbs increased considerably. This case differs from the previous in this very important feature, that there has been no remission and no relapse of symptoms. The symptoms, which started three and a half years ago, have steadily progressed, until the clinical picture you now see has developed. You will notice the difficulty he has in rising from a chair, and when he makes a step forward he requires the assistance of two sticks. There is a great deal of motor weakness in the leg; he cannot raise the legs, and you see a good deal of rigidity and stiffness. The gait of this patient is what is known as the spastic gait—a very common early symptom of disseminated sclerosis. There is no reason why one should diagnose disseminated sclerosis from the spastic paralysis unless there is evidence of disseminated lesion. Ascertain whether the lesions are disseminated by looking for other symptoms, such as nystagmus, instability, tremor in the movements of the arm, and atrophy of the optic disc with dimness of vision. This patient has presented no symptom of optic atrophy. The note says that he has slight nystagmus on looking to the left, though there is some reason to doubt whether this is so. Perhaps on more careful testing it might be made out. The finger-nose test shows that the lesion is clearly not confined to the upper lumbar or mid-dorsal region. There is a lesion causing some interference with the steady movement of the fingers and hand, and signs even of intention tremor. On examining the reflexes—and after all it is by examination of these that one is able to substantiate the existence of degenerative lesions of the fibres of the spinal cord—we find exaggerated tendon-jerks and extension of the great toe on testing the plantar reflexes, together with total absence of abdominal reflexes. There is not much rigidity, and the gait seems to be paretic rather than spastic. Before testing the plantar reflexes notice that the toes are in a condition of hyper-extension. The extensor plantar reflex is present on both sides. As a rule in these cases abdominal reflex is absent. The examination of the reflexes in this case therefore shows very active knee-jerks, double ankle-clonus, double extensor plantar response, and absent abdominal reflexes. So that in all respects this patient agrees with the first case with this difference, that instead of an ataxic and unsteady gait, he has a paretic gait.

Case 3.—This patient, 30 years of age, noticed, five years ago, a burning sensation in front of the left thigh, which soon passed away. Four years ago he noticed that if he cycled to any extent or fatigued himself with walking the left thigh became tired much more readily than the right, and if the fatigue were excessive the leg began to drag. One year ago numbness appeared in this leg. Nine months ago the symptoms, which had already been in existence for some years in the left thigh, made their appearance in the right, and unsteadiness of gait supervened. Four months ago some difficulty was experienced in emptying the bladder. If we compare the history of this case with the other two, we note a definite step by step method of onset, persisting over the better part of five years. First of all, early fatigue and dragging of the left foot, these symptoms passing next to the left leg, then numbness and uncomfortable sensations of the left leg; later on numbness and dragging of the right leg, then unsteadiness, and finally difficulty in passing water. At present he has difficulty in walking without the assistance of a stick, considerable instability, and a tendency to drag the left leg, both legs seeming to have a distinctly paretic character. At the same time there is a general appearance of unsteadiness in the upper part of the body, with a loss of sensation in the feet. Romberg's symptom is very marked. This patient is a contrast to the other cases in having paresis and instability of gait. Like the first patient, he has well-marked Romberg's symptom. There is no intention tremor when the test is applied, merely a certain amount of instability or awkwardness on movement of the hand. On testing the eyes there is slight nystagmoid jerkiness on looking to the right. The reflex conditions show active knee-jerks, the right very considerably in excess of the left, a suspicion of ankle-clonus on both sides, well-marked extensor plantar responses, and absent abdominal reflexes. In testing for the plantar reflexes, the finger or some blunt instrument should be carried along the arch of the foot slowly and with slight pressure, or across the balls of the toes. The absence of the abdominal reflexes in disseminated sclerosis is almost as important an observation as the presence of plantar reflexes of the extensor type.

Case 4.—Another patient, 27 years of age, has been ill for the better part of nine years. I show her to you because she presents what the other patients did not present—namely, the mental traits met with in dessiminated sclerosis. The patient has a facility of mind which is not uncommon in this disease, laughs readily and cries readily, and generally appears to be of a somewhat simple character, with some degree of dementia. The knee-jerks are brisk, the right rather in excess of the left. She has a little tremor in the right hand, but this symptom is not well marked. She has a spastic gait, considerable paraplegia, and instability when standing and walking. There is a little nystagmus on looking upward, but not on lateral movement, with optic atrophy.

### Differential Symptomatology.

If you take a rapid glance at the symptoms, you will notice that there are certain conditions which are almost constant in disseminated sclerosis and which should be regarded as definite evidence of the disease.

I.—(1) Paretic gait—sometimes spastic, sometimes purely paretic—a very common feature. (2) Ataxic or unsteady gait. Sometimes a combination of these two forms is met with, forming the (3) ataxic-spastic gait, which is so very characteristic of this disease.

II. Nystagmus.—This was present to some extent in all the cases.

III. Tremor.—I have not been able to show you to-day any cases of well-marked intention tremor so characteristic of this disease.

IV. Amblyopia and optic atrophy, in varying degree.

These are the main physical conditions which I have endeavoured to demonstrate in this series of cases.

### The Reflexes.

When we look to the reflexes the following are constant in disseminated sclerosis:—(1) Tendon-jerks, exaggerated. (2) Plantar response of the extensor type. (3) Abdominal reflexes absent.

### Differential Diagnosis from Hysteria.

There is one malady difficult in the early stages to distinguish from disseminated sclerosis, with a history on very similar lines to that disease, and characterised by curious anomalies of gait—namely, hysteria. The examination of the reflexes is the most certain way of distinguishing hysteria from sclerosis. In hysteria (1) the tendon-jerks are exaggerated; (2) the plantar reflex is never extensor in type, and it may be flexor or absent; (3) abdominal reflexes are present. Amblyopia occurs in both. Intention tremor is often of a functional nature. In the differential diagnosis between hysteria and disseminated sclerosis, the condition of the reflexes is therefore of great importance.

